# Validation of reaction norm breeding values for robustness in Australian sheep

**DOI:** 10.1186/s12711-023-00872-5

**Published:** 2024-01-05

**Authors:** Dominic L. Waters, Sam A. Clark, Daniel J. Brown, Samuel F. Walkom, Julius H. J. van der Werf

**Affiliations:** 1 School of Environmental & Rural Science, University of New England, Armidale, NSW 2351 Australia; 2 Animal Genetics and Breeding Unit, University of New England, Armidale, NSW 2351 Australia

## Abstract

**Background:**

There can be variation between animals in how stable their genetic merit is across different environments due to genotype-by-environment (G×E) interactions. This variation could be used in breeding programs to select robust genotypes that combine high overall performance with stable genetic ranking across environments. There have been few attempts to validate breeding values for robustness in livestock, although this is a necessary step towards their implementation in selection decisions. The objective of this study was to validate breeding values for the robustness of body weight across different growth environments that were estimated using reaction norm models in sheep data.

**Results:**

Using threefold cross-validation for the progeny of 337 sires, the average correlation between single-step breeding values for the reaction norm slope and the realised robustness of progeny across different growth environments was 0.21. The correlation between breeding values for the reaction slope estimated independently in two different datasets linked by common sires was close to the expected correlation based on theory.

**Conclusions:**

Slope estimated breeding values (EBV) obtained using reaction norm models were predictive of the phenotypic robustness of progeny across different environments and were consistent for sires with progeny in two different datasets. Selection based on reaction norm EBV could be used to increase the robustness of a population to environmental variation.

## Background

Livestock populations are often managed across a range of environments that vary between both locations and years. This can give rise to genotype-by-environment (G×E) interactions, which occur when the relative performance of a genotype depends on the environment in which it will exist. Genotype-by-environment interactions can be challenging in breeding programs, as it means that the genetic merit of a genotype can change depending on the location or year in which it will be used. However, G×E interactions also represent an opportunity, as they are a source of genetic variation from which genotypes that are more robust to environmental variation could be selected [1]. This genetic resource could become more valuable due to climate change, as farm environments are predicted to become more variable [2].

Genetic variation in robustness can be captured using reaction norm models, which regress the estimated breeding value (EBV) of individual genotypes across an environmental covariable (EC) that is representative of the environmental quality. A linear reaction norm estimates an intercept, which represents the overall EBV across all the EC values, and a slope, which captures how much the EBV of a genotype changes over the range of the EC and is inferred from the performance of relatives in different environments. An ideal genotype combines a high intercept (high overall performance) with a flat slope (robustness of performance) [3]. Variation in the slope can also be partitioned into the type of G×E interaction which underlies it; that is, heterogeneity of the genetic correlation (rank-type G×E), or heterogeneity of the genetic variance (scale-type G×E) [4]. Adjusting the slope EBV to account for scale-type G×E could help to identify sires that have a stable genetic ranking across environments [4].

A substantial body of research has demonstrated significant G×E interactions using reaction norm models in livestock [5–8], as well as their potential to increase the accuracy of phenotypic predictions relative to models that ignore G×E interaction [9–11]. It could also be useful to directly explore the predictive ability of reaction norm breeding values for robustness, as this would be an important step towards understanding the value of their inclusion in selection decisions.

Unlike conventional phenotypes, robustness cannot be directly measured on an individual animal. However, we can estimate the robustness of a sire’s genotype by recording progeny across a range of different EC levels. By estimating sire EBV for robustness using a training dataset, we can then examine their predictive ability in a test set. This type of cross-validation analysis has been used to validate reaction norm EBV for heat tolerance in dairy cattle [12], which eventually led to their implementation in selection programs [13]. The validity of robustness could also be tested if sires have progeny extensively recorded in two different datasets. The correlation between their EBV for robustness estimated independently in the two different datasets would provide a measure of their reliability.

The aim of this study was to explore the ability of linear reaction norm EBV to predict the robustness of the performance of progeny across environments using body-weight records collected on Australian Sheep. The EC was defined as the adjusted post-weaning growth rate of a contemporary group as a proxy for the quality of the environment under which growth performance was measured, and the analysed trait was post-weaning weight. Hence, robustness was captured as the change in EBV for post-weaning weight across different growth environments.

## Methods

### Data

Body weight at weaning (WWT, recorded between 50 and 120 days of age) and post-weaning (PWT, recorded between 120 and 329 days of age) was available for 34,584 lambs in the Australian Sheep CRC Information Nucleus Flock (INF) and Meat and Livestock Resource Flock (RF) [14]. The lambs were from Merino, Maternal (such as Border Leicester) or Terminal (such as Poll Dorset and White Suffolk) sires and Merino or first cross Maternal-Merino dams. A previous genomic analysis of the reaction norm for post-weaning weight in this dataset revealed significant genetic variation in the reaction norm slope [15]. The objective of the first part of the analysis was to test whether this genetic variation was predictive of the robustness of progeny performance using cross-validation and forward prediction.

Weaning and post-weaning body weights were available on another 344,888 pure-bred Merino lambs recorded by commercial stud breeders in a national evaluation system known as MERINOSELECT [16]. Many of the Merino sires recorded in the INF/RF data also had progeny recorded in this dataset. The objective of the second part of the analysis was to test whether the EBV for sires based on the reaction norm slope in the INF/RF data were consistent with the equivalent EBV estimated in the independent wider industry data.

#### Part 1. Cross-validation and forward prediction within the INF/RF dataset

As described in Waters et al. [15], the EC was calculated for 33,773 lambs as the best linear unbiased estimate (BLUE) of the post-weaning growth rate (PWGR) of each contemporary group (CG), where the CG consisted of a flock × year × management group combination. The management group consisted of animals which were subjected to the same management decisions within each flock-year, i.e., they were raised in the same paddocks and phenotyped at the same time. To be eligible for analysis, each lamb had to have a known sire and dam and a known birth-type and rear-type, to have at least 40 days between weaning and post weaning weights, to be a member of a contemporary group with at least 15 animals originating from at least three different sires, and to be within 4 standard deviations of the mean PWGR. The model to obtain the BLUE of the CG effects was:

1 $$\mathbf{y}=\mathbf{Xb}+{\mathbf{Z}}_{\mathbf{1}}\mathbf{a}+\mathbf{Qg}+\mathbf{e},$$

where $\mathbf{y}$ is the vector of PWGR records, $\mathbf{X}$ is an incidence matrix for the fixed effects $\mathbf{b}$, ${\mathbf{Z}}_{\mathbf{1}}$ is an incidence matrix relating the records to additive genetic effects $(\mathbf{a})$, $\mathbf{Q}$ is a matrix of the proportion of each animals’ genome originating from 39 breed-based genetic groups derived from their pedigree, $\mathbf{g}$ is the vector of random genetic group effects, and $\mathbf{e}$ is the vector of residual effects. Fixed effects included sex, birth-type by rear-type interaction, age at post-weaning (linear and quadratic), and CG. A random permanent environmental dam effect was also considered but was not significant based on a log-likelihood ratio test. The BLUE of the CG effects (249 CG in total) formed the EC variable in the reaction norm analysis, where it was standardised to have a mean of 0 and a variance of 1.

**Fig. 1:**
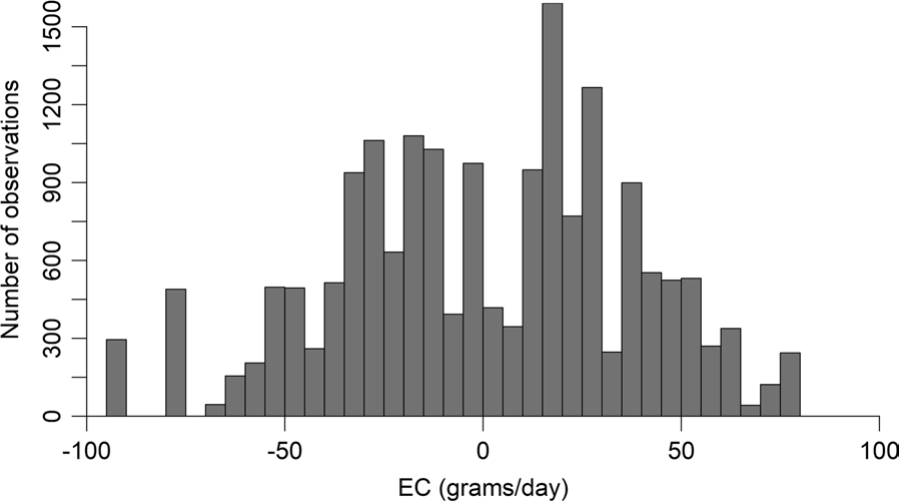
Distribution of 18,171 lambs across the mean-centred environmental covariables

Sires had to have at least 25 progeny across an EC range of at least 60 g/day to participate in the cross-validation and forward prediction analyses. This was to ensure reliable estimates of the change in performance across the EC. Of the 345 sires that met this criterion, 337 were only used between 2007 and 2011, while the remaining eight sires were used between 2012 and 2020. This was a result of the project design, as there were more locations tested between 2007 and 2011, so sires tended to have more progeny across different EC levels. Therefore, 18,171 lambs born between 2007 and 2011 were extracted for the validation analysis. The distribution of the lambs across the EC is given in Fig. 1.

Genomic data were available for 12,359 of the lambs, and consisted of 60,400 imputed single nucleotide polymorphisms (SNPs), as outlined in Waters et al. [15]. Genomic information was included in the reaction norm models using matrix $\mathbf{H}$, which combined the pedigree $(\mathbf{A})$ and genomic $(\mathbf{G})$ relationship matrices [17]. Matrix $\mathbf{A}$ was constructed using three generations of the pedigree (31,502 animals), while $\mathbf{G}$ was constructed following the first method proposed in VanRaden [18]. The two matrices were combined to form $\mathbf{H}$ in the MTG2 software [19] using a lambda value of 0.95.

##### Cross-validation

In the cross-validation, the progeny of each sire was split into one of three folds. To ensure each fold contained the maximum environmental range possible, progeny were ordered by EC within each sire. The first three progeny were then randomly assigned to a fold (1, 2 or 3), without replacement. This was repeated for the next three progeny along the EC, and so on for the remainder of their progeny. Only sires that had an environmental range of at least 60 g/day within each of the three folds and at least 25 progeny in total across the folds were considered in the cross-validation, leaving 337 sires of 14,612 lambs across 162 contemporary groups.

The single-step reaction norm models were trained on progeny from two of the folds, as well as the progeny of sires that did not meet the criteria for cross-validation (3559 lambs from 142 sires). The resulting EBV were then used to predict the realised progeny performance in the remaining fold (the test set) for the 337 validation sires. This was repeated for each of the three folds. A numerical summary for the three folds is given in Table 1, as well as the additional data from the progeny of ineligible sires used to train the models.

**Table 1:** Numerical summary of animals within each fold and the additional training data

Fold	1	2	3	Additional training
Number of animals	4876	4880	4856	3559
Number of genotyped animals	3331	3338	3294	2396
Number of contemporary groups	162	162	162	139
Post-weaning weight (kg)	43.1 ± 8.7	43.1 ± 8.8	43.1 ± 8.6	41.0 ± 9.1
EC (g/day)	0.86 ± 8.2	0.68 ± 38.2	0.76 ± 38.1	− 3.14 ± 32.9

Mean ± standard deviation is reported for post-weaning weight and the EC

The reaction norm model fitted was:

2 $$\mathbf{y}=\mathbf{Xb}+\mathbf{{{Z}}_{\mathbf{1}}}{{\mathbf{a}}_{\mathbf{0}}}+{{\mathbf{Z}}_{\mathbf{2}}}{{\mathbf{a}}_{\mathbf{1}}}+{{\mathbf{Z}}_{\mathbf{3}}}\mathbf{c}+\mathbf{Qg}+\mathbf{e},$$

where $\mathbf{y}$ is a vector of PWT phenotypes, $\mathbf{X}$ is an incidence matrix for the fixed effects $\mathbf{b}$, ${\mathbf{Z}}_{\mathbf{1}}$ and ${\mathbf{Z}}_{\mathbf{2}}$ are matrices relating records to the additive genetic effects for the intercept $({\mathbf{a}}_{\mathbf{0}})$ and slope $({\mathbf{a}}_{\mathbf{1}})$ respectively, with ${\mathbf{Z}}_{\mathbf{1}}$ containing 1s on the diagonal and ${\mathbf{Z}}_{\mathbf{2}}$ containing the EC value corresponding to each individual in $\mathbf{y}$ on the diagonal, ${\mathbf{Z}}_{\mathbf{3}}$ is an incidence matrix relating records to the permanent environmental dam effects $(\mathbf{c})$, which were estimated ignoring relationships between dams, $\mathbf{Q}$ is a matrix of the proportion of each animals’ genome originating from 39 breed-based genetic groups, $\mathbf{g}$ is the vector of random genetic group effects, and $\mathbf{e}$ is the vector of residual effects. Fixed effects included age at measurement (linear and quadratic), birth-type and rear-type interaction, sex, and contemporary group. The additive genetic variance of ${\mathbf{a}}_{\mathbf{0}}$ and ${\mathbf{a}}_{\mathbf{1}}$ was modelled according to $$\left[\begin{array}{c}{{\mathbf{a}}_{\mathbf{0}}}\\ {{\mathbf{a}}_{\mathbf{1}}}\end{array}\right]\sim\text{N}\left(0,\mathbf{H}\otimes \mathbf{K}\right)$$, where $$\mathbf{K} =\left[\begin{array}{ll}{ {\upsigma}}_{\text{a}0}^{2} & {{\upsigma}}_{\text{a}1\text{a}0} \\ {{\upsigma}}_{\text{a}0\text{a}1} & {{\upsigma}}_{\text{a}1}^{2} \end{array}\right]$$. The residual $(\mathbf{e})$ was modelled as a continuous function of the EC rather than using discrete intervals. An intercept $({\mathbf{e}}_{\mathbf{1}})$ and slope $({\mathbf{e}}_{\mathbf{2}})$ residual coefficient were used, such that $$\left[\begin{array}{c}{\mathbf{e}}_{\mathbf{1}}\\ {\mathbf{e}}_{\mathbf{2}}\end{array}\right]\sim\text{N}\left(0,\mathbf{I}\otimes \mathbf{E}\right)$$, where $$\mathbf{E}=\left[\begin{array}{ll}{ {\upsigma }}_{{\text{e}}_{1} }^{2} & {{\upsigma }}_{{{\text{e}}_{2}\text{e}}_{1}} \\ { {\upsigma }}_{{{\text{e}}_{1}\text{e}}_{2}} & {{\upsigma }}_{{\text{e}}_{2} }^{2} \end{array}\right]$$. The models were implemented using the MTG2 software [19].

In addition to the EBV for the intercept $({\mathbf{a}}_{\mathbf{0}})$ and slope $({\mathbf{a}}_{\mathbf{1}})$, scale corrected EBV for the slope $({\mathbf{a}}_{\mathbf{1}}^{\mathbf{*}})$ were also estimated using a genetic regression, which captures variation in the slope that is independent of the genetic correlation between the intercept and slope [4]:

3 $${\mathbf{a}}_{\mathbf{1}}^{\mathbf{*}}={\mathbf{a}}_{\mathbf{1}}-\frac{{{\upsigma }}_{{\text{a}}_{0}{\text{a}}_{1}}}{{{\upsigma }}_{{\text{a}}_{0}}^{2}}{\mathbf{a}}_{\mathbf{0}}.$$

##### Forward prediction

**Fig. 2:**
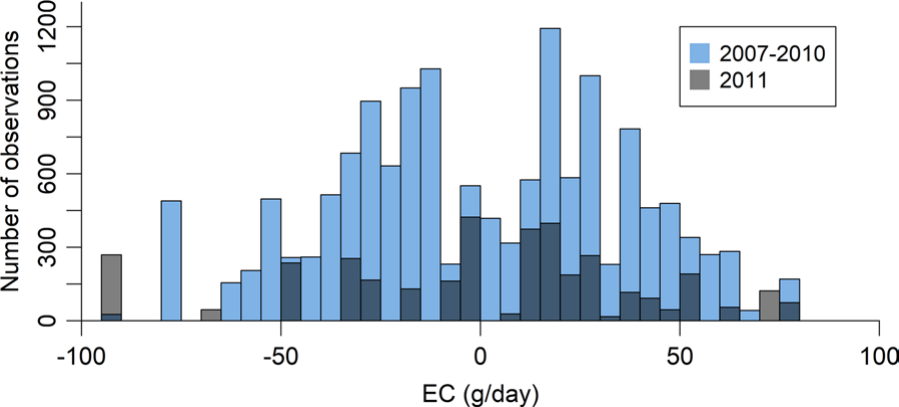
Distribution of lambs in 2007–2010 (blue) and 2011 (grey) across the mean-centred environmental covariables

In the forward prediction analysis, data from 14,521 lambs born in 2007 to 2010 were used to train the reaction norm model and predict the progeny performance of 88 sires with 3103 progeny born in 2011. Unlike the cross-validation, the progeny of validation sires were not used to train the reaction norm model, so they had a more distant relationship to the training population. The same reaction norm model as Eq. (2) was fit to the data. The distribution of the training and test data across the EC is given in Fig. 2.

##### Realised performance in the test set

The realised progeny performance across the EC in the test set was used to assess the predictive ability of the reaction norm EBV and was obtained as a linear random regression of the pre-adjusted phenotypes $({\mathbf{y}}^{\mathbf{*}})$ of the sire’s test progeny across the EC. The pre-adjustment factors were calculated in an animal model using the 18,171 lambs born between 2007 and 2011:

4 $$\mathbf{y}=\mathbf{Xb}+{\mathbf{Z}}_{\mathbf{1}}\mathbf{a}+{\mathbf{Z}}_{\mathbf{2}}\mathbf{c} +\mathbf{Qg}+\mathbf{e},$$

where $\mathbf{y}$ is the vector of PWT records, $\mathbf{X}$ is an incidence matrix for the fixed effects $\mathbf{b}$, which were the same as in Eq. (2), ${\mathbf{Z}}_{\mathbf{1}}$ is an incidence matrix relating the records to additive genetic effects $(\mathbf{a})$, and ${\mathbf{Z}}_{\mathbf{2}}$, $\mathbf{Q}$, $\mathbf{c}$, $\mathbf{g}$ and $\mathbf{e}$ were the same as in Eq. (2). The pre-corrected phenotypes $({\mathbf{y}}^{\mathbf{*}})$ were obtained as:

5 $${\mathbf{y}}^{\mathbf{*}}=\mathbf{y}-\mathbf{Xb} - {\mathbf{Z}}_{\mathbf{2}}\mathbf{c}-\mathbf{Qg}.$$

The pre-corrected phenotypes were then regressed across the EC for each sire within each test set using a linear random regression model:

6 $${\mathbf{y}}^{\mathbf{*}}=\mathbf{Xb}+{\mathbf{Z}}_{\mathbf{1}}{\mathbf{p}}_{\mathbf{0}}+{\mathbf{Z}}_{\mathbf{2}}{\mathbf{p}}_{\mathbf{1}}+\mathbf{e},$$

where $\mathbf{X}$, ${\mathbf{Z}}_{\mathbf{1}}$, ${\mathbf{Z}}_{\mathbf{2}}$ and $\mathbf{e}$ were the same as in Eq. (2), and ${\mathbf{p}}_{\mathbf{0}}$ and ${\mathbf{p}}_{\mathbf{1}}$ are the vectors of random sire regression coefficients for the intercept and slope, respectively. The variance of ${\mathbf{p}}_{\mathbf{0}}$ and ${\mathbf{p}}_{\mathbf{1}}$ was modelled according to $$\left[\begin{array}{c}{\mathbf{p}}_{\mathbf{0}}\\ {\mathbf{p}}_{\mathbf{1}}\end{array}\right]\sim\text{N}\left(0,\mathbf{I}\otimes \mathbf{K}\right)$$, where $$\mathbf{K}=\left[\begin{array}{ll}{ {\upsigma }}_{\text{p}0 }^{2} & { {\upsigma }}_{\text{p}1\text{p}0} \\ {{\upsigma }}_{\text{p}0\text{p}1 } & {{\upsigma }}_{\text{p}1 }^{2} \end{array}\right]$$ and $\mathbf{I}$ is an identity matrix (i.e., the model ignored the relationships between sires). The residual variance, $\mathbf{e}$, was modelled as a continuous function of the EC [i.e., the same as in Eq. (2)]. The regression coefficients ${\mathbf{p}}_{\mathbf{0}}$ and ${\mathbf{p}}_{\mathbf{1}}$ captured the overall phenotypic performance and robustness of each sire’s progeny in the test set, respectively. The Pearson correlation between the EBV for the sires in the training set and the realised progeny performance estimated from the test set was used to quantify the predictive ability of the EBV. The design of the cross-validation and forward prediction schemes is summarised in Table 2.

**Table 2:** Design of the cross validation and forward prediction analyses

Cross-validation	Training set	Test set
Run 1	Fold 2, 3 + additional data	Fold 1
Run 2	Fold 1, 3 + additional data	Fold 2
Run 3	Fold 1, 2 + additional data	Fold 3
Forward prediction	Years 2007–2010	Year 2011

#### Part 2. Validation of robustness EBV in industry data

Many of the Merino sires used in the INF/RF also had progeny recorded in the wider industry population, known as MERINOSELECT [16]. The aim of this analysis was to test whether reaction norm slope EBV estimated for Merino sires in the INF/RF data were consistent with the equivalent EBV estimated in the MERINOSELECT data. The analysis used pedigree data to model additive relationships because of both computational limitations and potential issues related to selective genotyping in the commercial data.

##### Data

There were 12,087 and 318,028 pure-bred Merino lambs in the INF/RF and MERINOSELECT databases, respectively, that had a known sire and dam and a known birth-type and rear-type, had at least 40 days between weaning and post-weaning weights, were a member of a contemporary group with at least 15 animals originating from at least three different sires and had a PWGR within 4 standard deviations of the mean. The INF/RF data differed from Part 1 of the analysis as it consisted of (1) only pure-bred Merino lambs, and (2) lambs born between 2007 and 2020. Contemporary groups in the MERINOSELECT data were only included if they contained at least one animal related to a sire or grandsire in the INF/RF data, leaving 277,060 lambs. Approximately 93% of the MERINOSELECT lambs were born between 2007 and 2020. The remaining 7% were born between 2000 and 2006 but were still included in the analysis because of their genetic relationship to the INF/RF dataset.

The same model as Eq. (1) was used to obtain a BLUE of PWGR for each CG using the joint INF/RF and MERINOSELECT datasets, except that an additional permanent environmental dam effect $(\mathbf{c})$ was fitted as it was significant based on a log-likelihood ratio test. This formed the EC for the reaction norm analysis, where it was standardised to have a mean of 0 and a variance of 1.

**Fig. 3:**
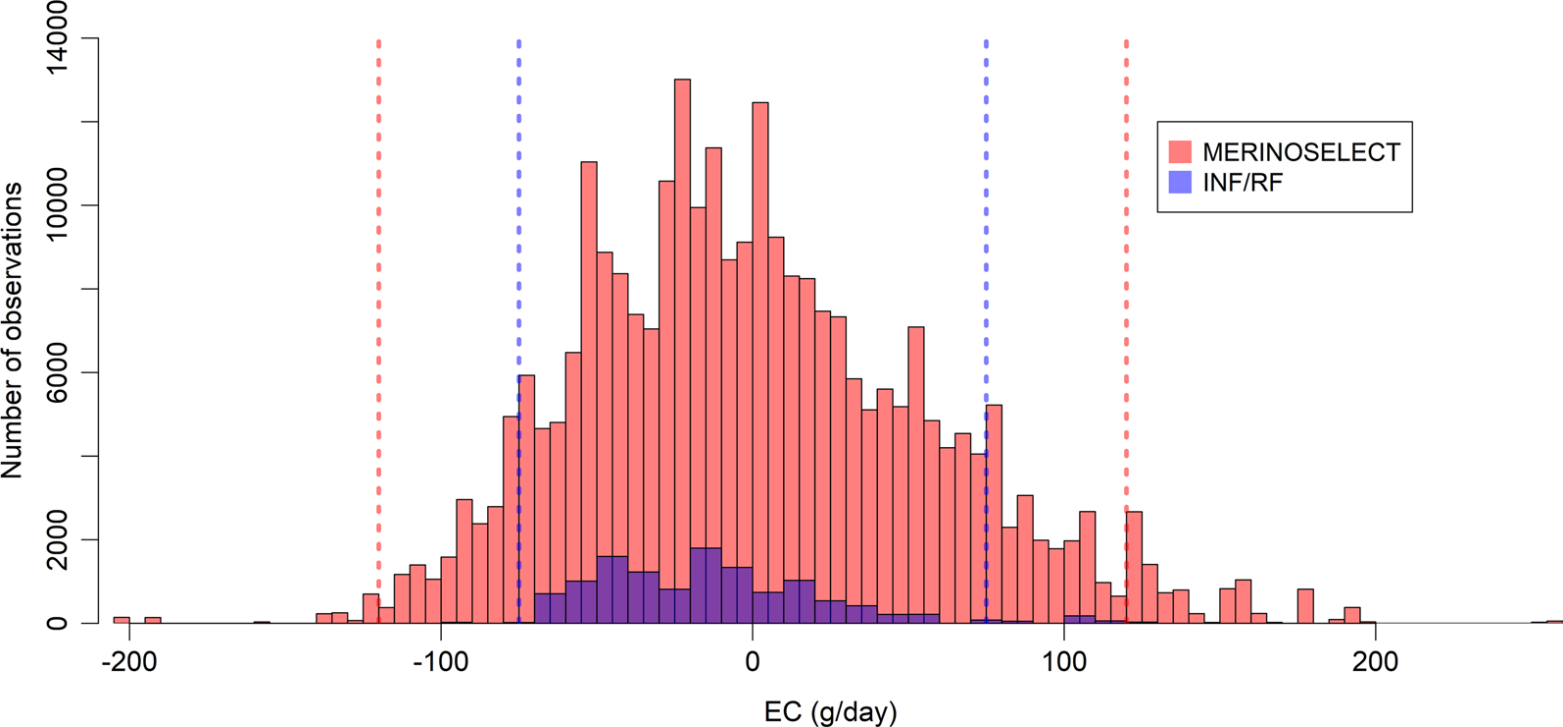
Distribution of lambs across the EC as a deviation from the overall mean environmental covariable in the MERINOSELECT (red) and INF/RF (purple) datasets. The vertical lines show the range of data used to fit the reaction norm models in the two datasets

After mean-centring, the EC effects ranged from − 99.2 to + 122.7 g/day in the INF/RF dataset and from − 200.9 to + 256.5 g/day in the MERINOSELECT dataset (Fig. 3). Only CG within ± 75 g/day (INF/RF), and ± 120 g/day (MERINOSELECT) of the mean were used in the reaction norm analysis, because small amounts of data at the ends of an EC can produce unreliable estimates in reaction norm models. This left 11,638 and 265,284 animals for the reaction norm analysis in the INF/RF and MERINOSELECT datasets, respectively. There were 253 common sires with 4729 and 23,150 direct progeny in the INF/RF and MERINOSELECT datasets, respectively. In total, 5291 animals in the INF/RF and 140,039 animals in the MERINOSELECT datasets were at least great-grand progeny of the common sires (i.e., their pedigree-based relationship was at least 0.125). A numerical summary of the final INF/RF and MERINOSELECT data is in Table 3.

**Table 3:** Numerical summary of animals in the INF/RF and MERINOSELECT, and mean values for traits and environmental covariate (EC) (± standard deviation)

	INF/RF	MERINOSELECT
Number of lambs	11,638	265,284
Number of sires with progeny data	615	6707
Number of dams	7070	150,962
Parities per dam	1.65 ± 0.89	1.76 ± 1.14
Number of contemporary groups	134	2335
Weaning age (days)	94.0 ± 9.8	95.2 ± 14.3
Post-weaning age (days)	262.0 ± 35.5	226.6 ± 40.8
Post-weaning growth period (days)	167.9 ± 36.9	131.4 ± 41.4
Weaning weight (kg)	24.5 ± 5.3	27.8 ± 6.6
Post-weaning weight (kg)	38.4 ± 8.04	40.4 ± 9.9
Post-weaning growth rate (g/day)	84.1 ± 39.4	98.7 ± 59.1
EC (g/day)	− 17.5 ± 29.8	− 3.6 ± 50.1

##### Reaction norms

Linear reaction norms were fit to the INF/RF and MERINOSELECT datasets independently to model how EBV for PWT changed across the EC using the ASReml 4.2 package [20]. ASReml 4.2 was used because it is very efficient at modelling large datasets with pedigree relationships (such as the MERINOSELECT data). The linear reaction norm models were as follows:

7 $$\mathbf{y}=\mathbf{Xb}+{\mathbf{Z}}_{\mathbf{1}}{\mathbf{a}}_{\mathbf{0}}+{\mathbf{Z}}_{\mathbf{2}}{\mathbf{a}}_{\mathbf{1}}+{\mathbf{Z}}_{\mathbf{3}}\mathbf{c}+{\mathbf{Z}}_{\mathbf{4}}\mathbf{m}+{\mathbf{Q}}_{\mathbf{g}}+\mathbf{e},$$

where $\mathbf{y}$, $\mathbf{X}$, ${\mathbf{Z}}_{\mathbf{1}}$, ${\mathbf{Z}}_{\mathbf{2}}$, ${\mathbf{Z}}_{\mathbf{3}}$, $\mathbf{Q}$, $\mathbf{b}$, ${\mathbf{a}}_{\mathbf{0}}$, ${\mathbf{a}}_{\mathbf{1}}$, $\mathbf{c}$, $\mathbf{g}$ and $\mathbf{e}$ were the same as in Eq. (2), and ${\mathbf{Z}}_{\mathbf{4}}$ is an incidence matrix relating records to the additive maternal genetic effects $(\mathbf{m})$. The variance of the additive maternal genetic effect was modelled according to: $\mathbf{m}\sim\text{N}\left(0,\mathbf{A}\otimes {{\upsigma }}_{\text{m} }^{2}\right)$. The covariance between ${ {\upsigma }}_{\text{m} }^{2}$ and all other variance components in the model was assumed to be zero. Additive maternal genetic effects were not fitted in the INF/RF model as they were not significant. The age of the dam at the time of the measurement of the lamb was fitted as a covariate (linear and quadratic) in the MERINOSELECT model. To account for heterogeneity within the Merino population, genetic groups were formed on a flock and time basis [16]. There were 252 and 432 genetic groups in the INF/RF and MERINOSELECT datasets, respectively.

The residual variance was estimated at four (− 75 to − 30 g/day, − 30 to 0 g/day, 0 to 30 g/day, 30 to 75 g/day) and six (− 120 to − 70 g/day, − 70 to − 30 g/day, − 30 to 0 g/day, 0 to 30 g/day, 30 to 70 g/day, 70 to 120 g/day) discrete classes along the EC for the INF/RF and MERINOSELECT dataset, respectively. The variance of the intercept and slope were modelled as follows: $$\left[\begin{array}{c}{\mathbf{a}}_{\mathbf{0}}\\ {\mathbf{a}}_{\mathbf{1}}\end{array}\right]\sim\text{N}\left(0,\mathbf{A}\otimes \mathbf{K}\right)$$ where $$\mathbf{K}=\left[\begin{array}{ll}{ {\upsigma }}_{\text{a}0 }^{2} & {{\upsigma }}_{\text{a}1\text{a}0} \\ {{\upsigma }}_{\text{a}0\text{a}1 } & {{\upsigma }}_{\text{a}1 }^{2} \end{array}\right]$$ and $\mathbf{A}$ is the pedigree relationship matrix. There were 24,431 and 532,181 animals in the pedigree for the INF/RF and MERINOSELECT datasets, respectively.

To compare estimates of the genetic variance and heritability between the datasets along the different levels of the EC, the genetic (co)variance between breeding values at different levels of the EC was obtained using $\mathbf{G}=\varvec{\Lambda }\mathbf{K}\varvec{\Lambda}^\mathbf{{\prime }}$, where $\mathbf{K}$ is the genetic (co)variance matrix for the intercept and slope, and $\mathbf{\Lambda }$ is a 100 × 2 matrix containing a vector of 1s for the intercept and a vector of standardised EC values ranging from the minimum to the maximum value of the EC. The heritability of PWT at a given EC level was then obtained by dividing the genetic variance at the EC level by the sum of the genetic, maternal genetic (if fitted), permanent environmental dam, and residual variance at the EC level. Scale corrected EBV for the slope $({\mathbf{a}}_{\mathbf{1}}^{\mathbf{*}})$ were estimated within each dataset using Eq. (3).

Sire EBV for the intercept $({\mathbf{a}}_{\mathbf{0}})$, slope $({\mathbf{a}}_{\mathbf{1}})$ and scale corrected slope $({\mathbf{a}}_{\mathbf{1}}^{\mathbf{*}})$ were compared between the two datasets using the Pearson product-moment correlation. This was compared to the expected correlation, which was calculated as the product of the average EBV accuracies of the two datasets. The accuracy of each reaction norm EBV was calculated as $\text{r}= \sqrt{1-\frac{\text{PEV}}{{{\upsigma }}_{\text{a}}^{2}}}$, where PEV is the prediction error variance of the EBV, and ${{\upsigma }}_{\text{a}}^{2}$ is the estimated genetic variance associated with either ${\mathbf{a}}_{\mathbf{0}}$ or ${\mathbf{a}}_{\mathbf{1}}$. The average accuracy $(\bar{\text{r}})$ of ${\mathbf{a}}_{\mathbf{0}}$ and ${\mathbf{a}}_{\mathbf{1}}$ was then obtained within each dataset using Fischer’s Z transformation, as untransformed correlations (and hence accuracies) are not normally distributed. The expected correlation for ${\mathbf{a}}_{\mathbf{0}}$ and ${\mathbf{a}}_{\mathbf{1}}$ was then ${\bar{\text{r}}}_{{{\mathbf{a}}_{\mathbf{0}}}_{\text{INF}/\text{RF}}}\times {\bar{\text{r}}}_{{{\mathbf{a}}_{\mathbf{0}}}_{\text{MERINOSELECT}}}$ and ${\bar{\text{r}}}_{{{\mathbf{a}}_{\mathbf{1}}}_{\text{INF}/\text{RF}}}\times {\bar{\text{r}}}_{{{\mathbf{a}}_{\mathbf{1}}}_{\text{MERINOSELECT}}}$, respectively. The expected correlation was calculated only for the intercept $({\mathbf{a}}_{\mathbf{0}})$ and slope $({\mathbf{a}}_{\mathbf{1}})$ EBV, as further investigation is required to derive the $\text{PEV}$ for ${\mathbf{a}}_{\mathbf{1}}^{\mathbf{*}}$. The expected correlation for ${\mathbf{a}}_{\mathbf{1}}$ served as an approximate expectation for ${\mathbf{a}}_{\mathbf{1}}^{\mathbf{*}}$.

## Results

### Part 1. Cross-validation and forward prediction within the INF/RF dataset

In the cross-validation analysis, the average correlation between EBV for the intercept and the realised overall progeny performance was high (0.67, Table 4). The EBV for the reaction norm slope and scale-corrected slope were moderately correlated with the realised robustness of progeny performance across the EC (0.18–0.21). Using forward prediction, the correlation between the reaction norm slope and the realised progeny robustness was higher than in the cross-validation analysis (0.28) but was lower for the intercept (0.53) and scale-corrected slope (0.12). Overall, the results show that the reaction norm EBV for the intercept, slope, and scale-corrected slope were predictive of the robustness of progeny performance across the EC.

The variance components estimated in each of the models are also reported in Table 5. Notably, the correlation between the intercept and slope was small and not significantly different from zero in all models. This suggests that there was little to no scale-type G×E interactions in the data. The relatively large standard errors for the correlation between the intercept and slope mean that the results for the scale-corrected slope $({\mathbf{a}}_{\mathbf{1}}^{\mathbf{*}})$ should be interpreted with caution.

**Table 4:** Correlations between reaction norm estimated breeding values (EBV) for sires and the realised regression of pre-corrected progeny phenotypes across the environmental covariable (EC)

Test set fold	${{\text{a}}}_{{0}}$ vs. ${\text{p}}_{{0}}$	${\text{a}}_{{1}}$ vs. ${\text{p}}_{{1}}$	${\text{a}}_{{1}}^{{*}}\; \text{vs}. \; {\text{p}}_{{1}}$
1	0.70	0.13	0.12
2	0.63	0.25	0.17
3	0.67	0.26	0.26
Average	0.67	0.21	0.18
Forward prediction (2011)	0.53	0.28	0.12

Cross-validation results (Fold 1, 2, and 3 and average) and forward prediction are reported

${\text{a}}_{0}$: sire EBV for intercept; ${\text{a}}_{1}$ sire EBV for slope; ${\text{a}}_{1}^{\text{*}}$: sire EBV for scale-corrected slope; ${\text{p}}_{{0}}$: realised progeny performance intercept; ${\text{p}}_{1}$: realised progeny peformance slope

**Table 5:** Variance components for the reaction norms models fitted to each fold in the cross-validation, the forward prediction (2007–2010), and all the data (18,171 lambs)

Data	${{\upsigma }}_{\text{a}0}^{2}$ (kg^2^)	${{\upsigma }}_{\text{a}1 }^{2}$ (kg^2^/SD EC)	${\text{r}}_{\text{a}0\text{a}1 }$	${{\upsigma }}_{{\text{a}}_{1} }^{2}/{{\upsigma }}_{{\text{a}}_{0} }^{2}$	g^2^	c^2^
Fold 1	8.40 (0.53)	0.66 (0.24)	0.10 (0.11)	0.08	40.92 (14.25)	1.99 (0.28)
Fold 2	7.29 (0.48)	0.67 (0.29)	0.08 (0.10)	0.09	40.77 (14.22)	2.29 (0.28)
Fold 3	8.11 (0.53)	0.66 (0.23)	− 0.01 (0.11)	0.08	44.97 (15.85)	1.98 (0.28)
2007–2010	7.94 (0.49)	0.69 (0.21)	0.09 (0.10)	0.09	39.31 (13.74)	2.19 (0.25)
All data	7.88 (0.44)	0.64 (0.18)	0.04 (0.09)	0.08	40.95 (14.29)	2.13 (0.22)

${{\upsigma }}_{\text{a}0 }^{2}$: additive genetic intercept; ${{\upsigma }}_{\text{a}1 }^{2}$: additive genetic slope; ${\text{r}}_{\text{a}0\text{a}1 }$: correlation between intercept and slope; g^2^: genetic group; c^2^: permanent environmental dam. Standard errors are given in parentheses

### Part 2. Validation in industry data

The variance components estimated using linear reaction norms for pure-bred Merinos in the INF/RF and MERINOSELECT datasets are in Table 6. The INF/RF had greater additive genetic variation in both the intercept and slope, while the ratio of slope to intercept variance was also greater in the INF/RF. This indicates that the G×E interactions were larger in the INF/RF than in the MERINOSELECT data. The genetic correlation between the intercept and slope was very similar, as well as the permanent environmental dam variance.

**Table 6:** Reaction norm variance components estimated in the INF/RF and MERINOSELECT (MS) data

Model	${{\upsigma }}_{{\text{a}}_{0} }^{2}$ (kg^2^)	${{\upsigma }}_{{\text{a}}_{1} }^{2}$ (kg^2^/SD EC)	${{\upsigma }}_{{\text{a}}_{1}}^{2}/{{\upsigma}}_{{\text{a}}_{0}}^{2}$	${\text{r}}_{\text{a}0\text{a}1 }$	g^2^	c^2^	m^2^
INF/RF	11.35 (0.96)	1.98 (0.88)	0.17	0.64	7.60 (2.04)	1.64 (0.33)	–
MS	8.06 (0.18)	1.00 (0.08)	0.12	0.60	4.47 (0.61)	1.28 (0.08)	0.95 (0.08)

Standard errors are given in parentheses

${{\upsigma }}_{\text{a}0 }^{2}$: additive genetic intercept; ${{\upsigma }}_{\text{a}1 }^{2}$: additive genetic slope; ${{\upsigma }}_{\text{a}1 }^{2}/{{\upsigma }}_{\text{a}0 }^{2}$: ratio of intercept and slope variance; ${\text{r}}_{\text{a}0\text{a}1 }$: correlation between intercept and slope; g^2^: genetic group; c^2^: permanent environmental dam; m^2^: maternal variance

**Fig. 4:**
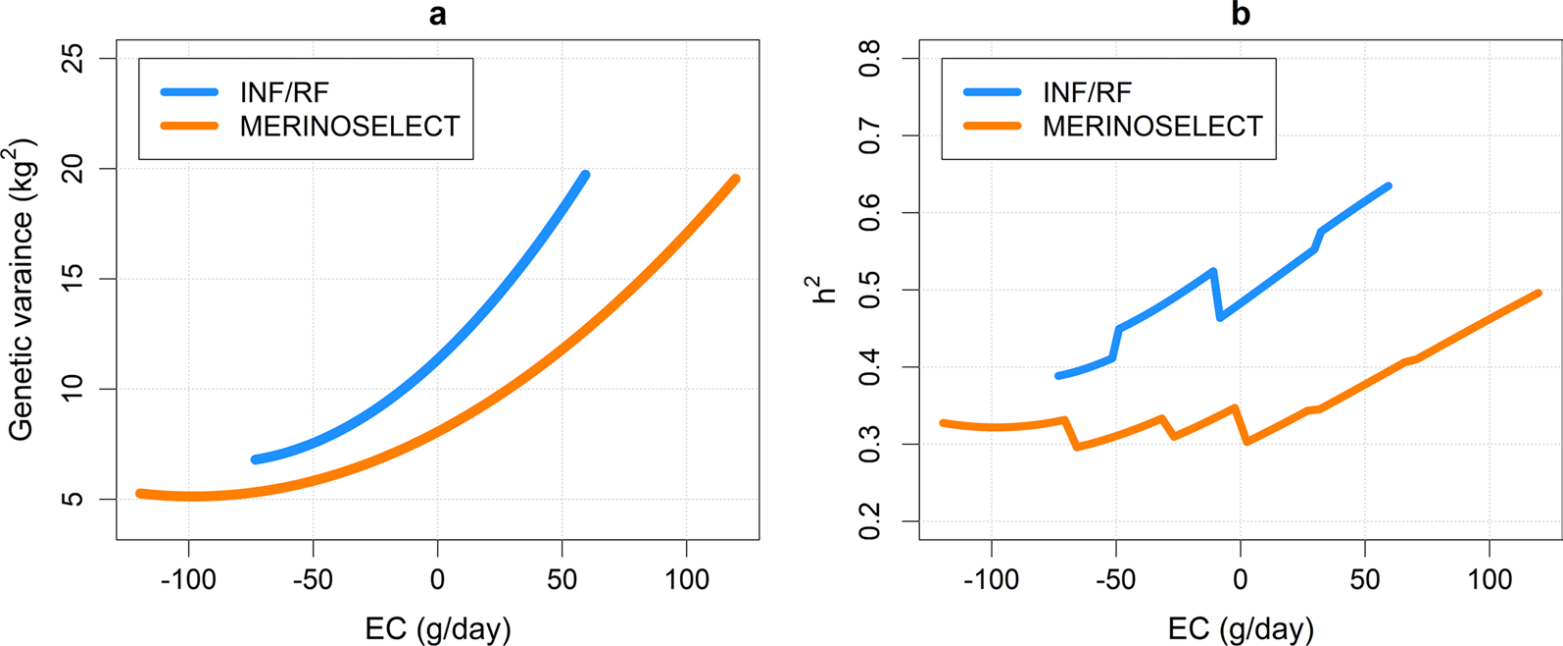
Genetic variance (**a**) and heritability (**b**) across the environmental covariables in the MERINOSELECT and INF/RF datasets

The genetic variance and heritability were consistently higher across the EC in the INF/RF data compared to the MERINOSELECT data (Fig. 4). However, the pattern of increase along the EC was very similar, indicating large scale-type G×E interactions in both datasets.

Using all sires with progeny in both datasets, the correlations for the reaction norm slope and scale-corrected slope EBV were 0.22 and 0.13, respectively (Table 7), while the correlation between intercept EBV was higher (0.40). The correlations for all EBV increased when only sires with either the most progeny or the most contemporary groups were considered. The correlations for the slope were only slightly lower than those expected based on the accuracy of the EBV (Table 7). Since the expected correlation represents the upper bound given the accuracy of the EBV, we can conclude that the EBV for robustness were consistent across the two independent datasets.

**Table 7:** Correlation of reaction norm estimated breeding values (EBV) for intercept $({\text{a}}_{0})$, slope $({\text{a}}_{1})$ and scale-corrected slope $({\text{a}}_{{1}}^{{*}})$

EBV	a	b	c
${\text{a}}_{0}$	0.40 (0.67)	0.62 (0.76)	0.64 (0.76)
${\text{a}}_{1}$	0.22 (0.32)	0.33 (0.37)	0.31 (0.38)
${\text{a}}_{1}^{\text{*}}$	0.13	0.32	0.34
Number of sires	253	65	62

The expectation of the correlation based on the accuracy of the EBV is given in parentheses

a: sires with direct progeny in both datasets; b: sires with at least 45 progeny in the MERINOSELECT and 15 progeny in the INF/RF; c: progeny in at least four CG in both datasets

## Discussion

The aim of this study was to validate breeding values for robustness that were estimated using reaction norm models in Australian sheep. The results showed that reaction norm EBV for the slope, which can be interpreted as ‘robustness EBV’, were predictive of the robustness of phenotypes of progeny across a range of environments. In addition, the reaction norm EBV were consistent across different datasets. Therefore, reaction norm EBV could be used to select sires that yield lambs that have a weight gain that is more robust to environmental variation.

The closest comparable validation analysis of robustness to our study was a study that examined heat tolerance in dairy cattle [12], and in which the robustness of milk, fat and protein yields to a temperature-humidity index was calculated for each cow using a linear random regression model to estimate a slope while ignoring genetic relationships. The robustness of sires was then obtained as the combination of their daughters’ slopes, and heat tolerance EBV were estimated using the slopes for genotyped sires and cows as phenotypes in a genomic best linear unbiased prediction (GBLUP) model. Using forward prediction with a reference population of 2300 Holstein sires, the correlation between heat tolerance EBV and the realised heat tolerance ranged from 0.28 to 0.31 for the first parity. The forward prediction strategy in our study was close to their validation structure and yielded a similar correlation of 0.28, although our study included substantially fewer sires (337 sires vs. 2300 sires) and measured animals (18,171 lambs vs. 366,835 cows). From this perspective, the results of the current study are promising, as heat-stress EBV are now successfully applied in dairy breeding programs [13].

To evaluate the correlations obtained in the cross-validation analysis, 337 sires with 43 progeny each were simulated across an EC, assuming the same variance components as estimated for all the data (Table 5). The simulation assumed that sires were unrelated and were randomly mated to dams distributed across the EC. Across 100 runs with threefold cross-validation, the average correlation between training and test sets was 0.68 for the intercept, and 0.12 for the slope and scale-corrected slope using random regression. This was consistent with the results in Table 4, although the empirical correlation was slightly higher for the reaction norm slope EBV. This was probably because we modelled the relationships between animals using genomic information, while the simulation assumed that sires were unrelated. This gives further reassurance that the results in the current study demonstrate the potential of selecting reaction norm EBV to improve robustness.

The correlations between reaction norm EBV estimated in the INF/RF and MERINOSELECT datasets were slightly lower than expected by theory. This was expected, as the theoretical correlation assumed that the genetic correlation between the datasets was 1. The deviations from theory were consistent with a genetic correlation between datasets of approximately 0.60–0.85 for the intercept, and 0.70–0.90 for the slope (e.g., for the slope in Table 7 scenario (b), 0.37 × 0.90 = 0.33, which was the realised correlation). This could be reasonable given that the MERINOSELECT models were trained on lambs across a much wider range of EC values (i.e., larger differences between environments), which could result in G×E interactions occurring between the datasets. Therefore, the analysis demonstrated that reaction norm EBV for robustness in PWT to different growth environments were consistent across two independent datasets and could be used for selection.

The correlation of reaction norm EBV between the INF/RF and MERINOSELECT datasets was higher for sires with (1) the most progeny, and (2) the most contemporary groups in both datasets. This highlighted the importance of data structure to accurately capture genetic variation in robustness. Sires require progeny across a wide range of EC values to estimate the reaction norm slope accurately [21]. Therefore, the success of breeding for robustness will likely depend on the structure of data available to estimate it accurately. If robustness is to be considered in genetic evaluations, breeders should be encouraged to ensure even stronger genetic linkage across years and locations. Alternatively, genomic EBV for robustness could be supplied using an appropriately designed reference population, such as the INF/RF used in this study. Selection based on genomic EBV for traits only measured within the INF/RF (such as traits related to meat eating quality) has yielded significant responses in the Australian sheep flock (Sheep Genetics, 2022), so a similar response could conceivably be gained for robustness. However, the INF/RF data had a much narrower range of EC values compared to the MERINOSELECT data (Fig. 1), which could limit its ability to predict robustness in PWT to different growth environments. The results of this study also indicated the existence of G×E interactions occurring between the research and industry datasets. The use of industry data (i.e., MERINOSELECT) to predict robustness in industry animals is a sensible solution to this problem.

Another way of estimating breeding values for robustness would be to reconsider the EC used to regress EBV on. While we used the adjusted post-weaning growth rate of the contemporary groups, other EC could be derived from measured environmental variables. This approach appears to work well for traits for which the time during which a small number of environmental factors are important in influencing the phenotype, is short. For instance, Nel et al. [7] found significant changes in the genetic variation of lamb survival as a function of a cold stress EC, which was calculated as a function of temperature, wind, and rainfall. Acute cold stress was an ideal EC to regress on, as it is a critical factor that influences neonatal lamb survival [22, 23]. The regression of milk production on a temperature-humidity index in dairy cattle is also ideal for these reasons [24]. In contrast, the post-weaning weight of a lamb is affected by many interacting environmental factors over a 6-month period, which makes it hard to identify and parameterise the relevant environmental descriptors. A vegetation index and a temperature-humidity index were recently used in a reaction norm analysis of body weight in Australian sheep [25]. While both EC captured significant G×E interactions, it could be challenging to combine both variables into a single genetic evaluation. In contrast, the adjusted post-weaning growth rate of a contemporary group automatically indexes the environmental factors into a single value with a practical interpretation; it captures the unit change in genetic merit of an individual for every unit change in growth environment. It also does not require any additional investment in data capture since it is derived from phenotypes that are routinely collected in breeding programs. Previous research across different livestock species and traits have identified growth rate as a suitable EC [6, 26, 27]. The results of our study demonstrated that the differences in robustness of sire EBV to a post-weaning growth EC were repeatable, meaning that it captured meaningful G×E interactions and could be used for selection.

The scale-corrected slope EBV represented slope variation that was independent of the genetic correlation with the intercept. This meant that they captured slope variation that was available for selection independent of the overall performance. In the across-dataset analysis, the genetic correlation between intercept and slope was reasonably large (0.60–0.64), which meant that the scale correction (Eq. 6) had a large effect on the slope EBV. When considering the sires with the best distribution of progeny across the EC, the scale corrected EBV were as highly correlated between datasets as the raw slope values. This indicated a significant genetic variation in robustness that could be selected alongside overall performance in PWT.

In the validation within the INF/RF data, the scale-corrected slope did not improve the predictive ability relative to the uncorrected reaction norm slope. However, the genetic correlation between the intercept and slope across all the data was 0.04, implying that only 0.16% (0.04^2^ × 100) of the genetic variation in the slope was due to scale-type G×E [28]. The standard errors for the genetic correlation were also large relative to the size of the correlation. This means that the breeding values for the scale-corrected slope were (1) not very different to the uncorrected breeding values, and (2) were corrected with low precision. Therefore, the results for the scale-corrected slope should be treated with caution. The smaller correlation between the intercept and slope found in this analysis could indicate that modelling the residual as a continuous function (Part 1) was able to adjust for the changes in phenotypic variance better than the discrete classes used in the across-dataset (Part 2) analysis, as suggested by Lillehammer et al. [29]. This should also be interpreted with caution, as the two analyses used different data (i.e., different breed compositions, flocks, and years) which could explain the difference in correlation.

## Conclusions

Differences between sires in their progeny performance across environments can be predicted using reaction norm models. Selection based on EBV from reaction norm models could be used to improve the robustness of weight gain in Australian sheep to different growth environments. The success of selection for robustness will depend on the availability of appropriate data structures to estimate it. If selection for robustness is to be implemented, sires need to be used across a wide range of environments to accurately estimate their EBV.

## Data Availability

The data are owned by Meat and Livestock Australia and Sheep Genetics Australia and access to the data can be negotiated by request.
