# Correction: Validation of reaction norm breeding values for robustness in Australian sheep

**DOI:** 10.1186/s12711-024-00877-8

**Published:** 2024-01-25

**Authors:** Dominic L. Waters, Sam A. Clark, Daniel J. Brown, Samuel F. Walkom, Julius H. J. van der Werf

**Affiliations:** 1 School of Environmental and Rural Science, University of New England, Armidale, NSW 2351 Australia; 2 Animal Genetics and Breeding Unit, University of New England, Armidale, NSW 2351 Australia

**Correction: Genetics Selection Evolution (2024) 56:4 **10.1186/s12711-023-00872-5

After publication of this original article [1], we noticed that an error was introduced in Eq. (2) page 3 which should be:

2 $$\mathbf{y}=\mathbf{X}\mathbf{b}+{\mathbf{Z}}_{1}{\mathbf{a}}_{\mathbf{0}}+{\mathbf{Z}}_{\mathbf{2}}{\mathbf{a}}_{\mathbf{1}}+{\mathbf{Z}}_{\mathbf{3}}\mathbf{c}+\mathbf{Q}\mathbf{g}+{\mathbf{e}},$$

instead of :

$$\mathbf{y}=\mathbf{X}\mathbf{b}+{P\mathbf{Z}}_{\mathbf{1}}{\mathbf{a}}_{\mathbf{0}}+{\mathbf{Z}}_{\mathbf{2}}{\mathbf{a}}_{\mathbf{1}}+{\mathbf{Z}}_{\mathbf{3}}\mathbf{c}+\mathbf{Q}\mathbf{g}+{\mathbf{e}}.$$
